# Modelling the impact of alcohol consumption on cardiovascular disease mortality for comparative risk assessments: an overview

**DOI:** 10.1186/s12889-016-3026-9

**Published:** 2016-04-28

**Authors:** Jürgen Rehm, Kevin D. Shield, Michael Roerecke, Gerrit Gmel

**Affiliations:** Centre for Addiction and Mental Health, 33 Russell Street, Toronto, M5S 2S1 ON Canada; Addiction Policy, Dalla Lana School of Public Health, University of Toronto (UofT), 155 College Street, 6th FL, Toronto, M5T 3 M7 ON Canada; Department of Psychiatry, Faculty of Medicine, UofT, 250 College Street, 8th FL, Toronto, M5T 1R8 ON Canada; Faculty of Medicine, Institute of Medical Science, UofT, Medical Sciences Bldg, 1 King’s College Circle, Room 2374, Toronto, M5S 1A8 ON Canada; Institute of Clinical Psychology and Psychotherapy, Technische Universität Dresden, Chemnitzer Str. 46, Dresden, 01187 Germany; School of Electrical Engineering and Telecommunications, The University of New South Wales, High Street, Kensington, NSW 2052 Australia; Implant Systems Group, National Information and Communications Technology Australia, Sydney, Australia 13 Garden Street, Eveleigh, NSW 2015 Australia

**Keywords:** Alcohol consumption, Cardiovascular disease, Ischaemic heart disease, Ischaemic stroke, Mortality, Global health

## Abstract

**Background:**

Although alcohol consumption has long been considered as a risk factor for chronic disease, the relationship to cardiovascular disease (CVD) is complex and involves at least two dimensions: average volume of alcohol consumption and patterns of drinking. The objective of this contribution was to estimate the burden of CVD mortality caused by alcohol consumption.

**Methods:**

Risk assessment modelling with alcohol-attributable CVD mortality as primary outcome. The mortality burden of ischaemic heart disease (IHD) and ischaemic stroke (IS) attributable to alcohol consumption was estimated using attributable-fraction methodology. Relative Risk (RR) data for IHD and IS were obtained from the most comprehensive meta-analyses (except for Russia and surrounding countries where alcohol RR data were obtained from a large cohort study). Age-group specific RRs were calculated, based on large studies. Data on mortality were obtained from the World Health Organization’s Global Health Estimates and alcohol consumption data were obtained from the Global Information System on Alcohol and Health. Risk of former drinkers was modelled taking into account global differences in the prevalence of sick quitters among former drinkers. Alcohol-attributable mortality estimates for all other CVD causes except IHD and IS were obtained from the 2014 Global Status Report on Alcohol and Health.

**Results:**

An estimated 780,381 CVD deaths (441,893 and 338,490 CVD deaths among men and women respectively) were attributable to alcohol consumption globally in 2012, accounting for 1.4 % of all deaths and 26.6 % of all alcohol-attributable deaths. This is in contrast to the previously estimated 1,128,273 CVD deaths attributable to alcohol consumption globally, and represents a decrease of 30.8 % in alcohol-attributable CVD mortality and of 10.6 % in the global burden of all alcohol-attributable deaths.

**Conclusions:**

When the most comprehensive and recent systematic reviews and meta-analyses are taken as bases, the net impact of alcohol consumption on CVD is lower than previously estimated.

**Electronic supplementary material:**

The online version of this article (doi:10.1186/s12889-016-3026-9) contains supplementary material, which is available to authorized users.

## Background

Although alcohol consumption has long been considered as a risk factor for cardiovascular disease (CVD) [1], the effect of alcohol consumption on the risk of these diseases is complex [2, 3], as there are beneficial and detrimental effects depending on volume and patterns of alcohol consumption. The consumption of alcohol is causally related to several major CVD categories: hypertensive diseases [4]; ischaemic heart disease (IHD) [2, 5, 6]; cardiac arrhythmias [7]; ischaemic and haemorrhagic strokes [8]; and alcoholic cardiomyopathy (which is 100 % attributable to alcohol) [9]. Alcohol consumption impacts on CVD via multiple pathways, not the least of which is via the causal effect on the risk of high blood pressure [4, 10]. This is especially consequential, as high blood pressure is the most important overall risk factor for the global burden of disease [11].

The consumption of alcohol has a positive or negative impact on the risk of CVDs depending on the volume of alcohol consumed and the way in which alcohol is consumed [2, 3, 10, 12]. Furthermore, the effects of alcohol on the risk of CVDs can be immediate [3, 13–16] as well as longer-lasting [3, 17].

Given the complex relationship between alcohol consumption and the risk of CVDs, the quantification of the health burden of CVDs caused by alcohol consumption is difficult, especially for IHD and ischaemic stroke (IS), where the volume of alcohol consumed, the patterns of alcohol consumption and the beneficial effects of alcohol have to be taken into consideration. Recent results of such quantification models can be observed in the comparative risk assessments of the Global Burden of Disease studies [11, 18] and in the monitoring efforts of the World Health Organization (WHO) in the Global Status Reports on Alcohol and Health (GSRAH) ([19]; for the underlying alcohol Relative Risk (RR) functions used in the GSRAH, see [20]).

However, recently published meta-analyses on alcohol consumption and the risks for CVDs in general and IHD in particular suggest that new algorithms are required for modelling the effects of alcohol on ischaemic diseases [21–23]. This article will detail the reasoning behind the use of these new algorithms, and apply the resulting calculations to data from the recently published WHO GSRAH which appeared in 2014 and contained estimates for 2012 [19]. Thus, the main objective of this article is to estimate the impact of alcohol consumption on CVD mortality, both globally and regionally, based on the newest systematic reviews and meta-analyses.

## Methods

The burden of IHD and IS attributable to alcohol consumption was modelled using an attributable-fraction approach, where an attributable fraction is defined as the burden of disease that would not be present without the exposure under consideration [24]. In the case of alcohol consumption as the exposure, this counterfactual scenario [25] has usually been operationalized by assuming lifetime abstention for the respective population [26].

### Exposure and mortality data

The calculation of the Alcohol-Attributable Fraction (AAF) requires the combination of data on (i) the prevalence of different drinking statuses (current drinker, former drinker, lifetime abstainer), (ii) average consumption among current drinkers, (iii) the prevalence of people who are heavy episodic drinkers, and (iv) *per capita* consumption of alcohol. Exposure estimates for drinking status by sex, age (age groups: 15 to 34 years of age, 35 to 64 years of age, and 65 plus years of age; as usual for comparative risk assessments, no consumption was assumed for people under 15 years of age) and country, as well as data on the average drinking level among drinkers and the prevalence of regular and irregular heavy drinking occasions, were obtained from the Global Information System on Alcohol and Health (GISAH) (for a brief description of data sources, see [27]; http://apps.who.int/gho/data/node.main.GISAH). As alcohol is a legal substance, there are sales and/or production, export and import data for every country. However, as the prevalence of sick quitters (i.e. those who stopped consuming alcohol because of health reasons) among former drinkers may be different based on the country (e.g., in Thailand, where lifetime abstention rates are high, the prevalence of sick quitters among former drinkers is likely lower than that of European countries such as Russia), modifications were made to the prevalence of former drinkers when modelling the burden of IHD and IS attributable to alcohol consumption (see below). Total *per capita* consumption of alcohol data were obtained from the GISAH (these estimates were also the basis for exposure estimates used in the WHO’s 2014 GSRAH [19]).

Data on mortality by sex, age, cause of death and country for the year 2012 were obtained from the WHO Global Health Estimates (http://www.who.int/healthinfo/global_burden_disease/en/).

### Risk relations and calculating the alcohol-attributable fraction (AAF)

In the WHO 2014 GSRAH, the AAFs for most causes of death were calculated for each cardiovascular cause of death separately by sex, age (age groups: 15 to 34 years of age, 35 to 64 years of age, and 65 plus years of age), and country. To calculate the AAF, the Relative Risks (RR) for CVD mortality for different groups in comparison to lifetime abstainers were multiplied with the prevalence of these groups. The groups used were former and current drinkers, where the latter were modelled continuously based on their average drinking per day level. This modelling strategy has become standard in comparative risk assessments for alcohol, both for the Global Burden of Disease Studies [12] and the WHO GSRAH.

### Application of the alcohol-attributable fractions to causes of death

Alcohol attributable deaths by sex, age, and CVD cause of death categories were obtained for each country by multiplying the respective AAFs with the absolute numbers of death in the respective categories as defined by the various categories listed above. For instance, if there had been 1000 ischemic heart disease deaths for women ages 35–64 in a country, and if the AAF for this sex, age and cause of death category was 14 % for this country, the absolute number of deaths was 140.

### Uncertainty

As with all comparative risk analyses for alcohol, uncertainty was estimated with Monte Carlo methodology, described in more detail in Additional file 1: Web Appendix 1.

### Differences of the current analyses to the WHO 2014 GSRAH and their scientific basis

We re-conducted the analyses from the WHO’s 2014 GSRAH using data on risk relations (i.e., RR functions) from new meta-analyses for IHD (for an overview of the changes, see Table 1), and for IS (where the only changes were in modelling the “sick quitter effect” [28, 29] and age-specific alcohol RR functions).

**Table 1:** Differences in modelling ischaemic heart disease mortality between the 2014 GSRAH and this paper

	New algorithms	Algorithms from GSRAH
Risk relation curve up to 100 g/day	based on [5]; RR ≥ 1 after 60 g/day	based on [5]; RR ≥ 1 after 60 g/day
Risk relation curve for 100 g+/day	Yes, modelled based on [21, 22]	Not included, set to 1
Impact of binge drinking in persons who drink on average less than 60 g/day	Yes [6]	Yes [6]
Modelling the sick quitter effect in former drinkers	Only for high-income countries where the literature of alcohol RRs for former drinkers originated (see Additional file 1: Web appendix 2) [36]	For all countries, ex-drinkers were modelled with the increased risk of all-cause mortality RRs
Age-specific risk relations	For global estimates and for Russia and surrounding countries; the latter RRs were based on specific Russian data [34, 35]. Details are described in Additional file 1: Web-Appendix 4.	Only for global

The following information was used to model the relationship between alcohol consumption and both IHD and IS:

- 1) For both of the included ischaemic disease categories, namely IHD and IS, there is evidence of a beneficial effect (quantification for IHD: [5, 12]; for discussion: [30, 31]; for IS: [8]).
- 2) For both IHD and IS, there is evidence that with either irregular or regular heavy drinking occasions, the beneficial effect disappears [2, 6, 8, 14, 16, 32], which must be taken into account even for light to medium drinkers.
- 3) For both IHD and IS, as well as for CVDs in general, there is evidence that very heavy drinking occasions (e.g., by people with alcohol dependence) are associated with detrimental effects [21, 23, 33–35].
- 4) The effect of former drinking is detrimental according to meta-analyses [8, 36, 37]; however, most of these meta-analyses originated in high-income countries, where drinking over a lifetime is the norm [19], and where a substantial proportion of people in later adulthood quit drinking for health reasons [38–40]. As a consequence, we applied the specific RR for former drinkers [36] to the total population of former drinkers only in these regions, and artificially capped the prevalence of former drinkers for other regions (see Additional file 1: Web-Appendix 2 for details and Additional file 1: Web Appendix 3 for the countries within each region).
- The reasoning for capping was mainly because we wanted to be conservative and restrict the detrimental impact of former drinking to region, where we actually have evidence of the phenomenon (but see [41]). Unless we find better evidence for increased health risks of former drinkers due to alcohol in low- to mid income countries, this seems to be the cautious choice. This decision is also in line with the general rule of the comparative risk assessments for alcohol to always choose the more conservative option (e.g., [18]).
- 5) Additionally, the impact of alcohol on all-cause mortality and on IHD and IS is age-specific [42]. Accordingly, the alcohol risk for IHD and IS was modelled based on age-specific RRs estimated based on the observed effect modification of age on the relationship between alcohol and IHD and IS [6, 8].

The methodology described above was used for all countries except for Russia and surrounding countries, in which case we used country-specific RRs based on a large epidemiological study from Zaridze and colleagues [35]. The RRs provided by Zaridze and colleagues have been recently corroborated by a large cohort study [34], and are in line with natural experiments such as the Gorbachev reform [43], where a marked reduction of alcohol consumption was associated with a marked reduction of cardiovascular mortality [44]. This shows that for Russia and surrounding countries, the detrimental effects of regular and irregular heavy drinking occasions by far outweigh the beneficial effects. Additionally, for these countries, the risk relations were based on [34] (see Additional file 1: Web Appendix 4 for details).

The modelling strategy for IHD compared to that used in the 2014 GSRAH is summarized in Table 1. For IS mortality, we used the alcohol RRs from the meta-analyses of Patra and colleagues (for risk curves see [8]); for people with heavy drinking occasions and an average volume of alcohol consumption of up to 60 g of pure alcohol per day, we set the RR to 1 (for the rationale, see [14] and above).

This modelling approach is in line with biological pathways, where regular light to moderate drinking is associated with more favourable blood lipid profiles, fibrinogen levels, inhibition of platelet activation, and anti-inflammatory effects [3, 17, 45]; irregular binge episodes, which are characteristic of the Russian style of drinking, lead to opposite effects, and an increase in hypertension and arrhythmias [3, 46–48]).

The resulting alcohol-attributable burden estimated using data from the most recent and comprehensive evidence were compared to the results from the last WHO comparative risk assessment for alcohol consumption for the year 2012 presented in the 2014 GSRAH [19].

### Ethical approval

The study was approved by the Research Ethics Board of the Centre for Addiction and Mental Health, Toronto, Canada, as part of the Comparative Risk Analyses for alcohol. All the data sources used can be obtained by the authors upon request.

## Results

### Relative risk relations between alcohol consumption and ischaemic diseases

**Fig. 1:**
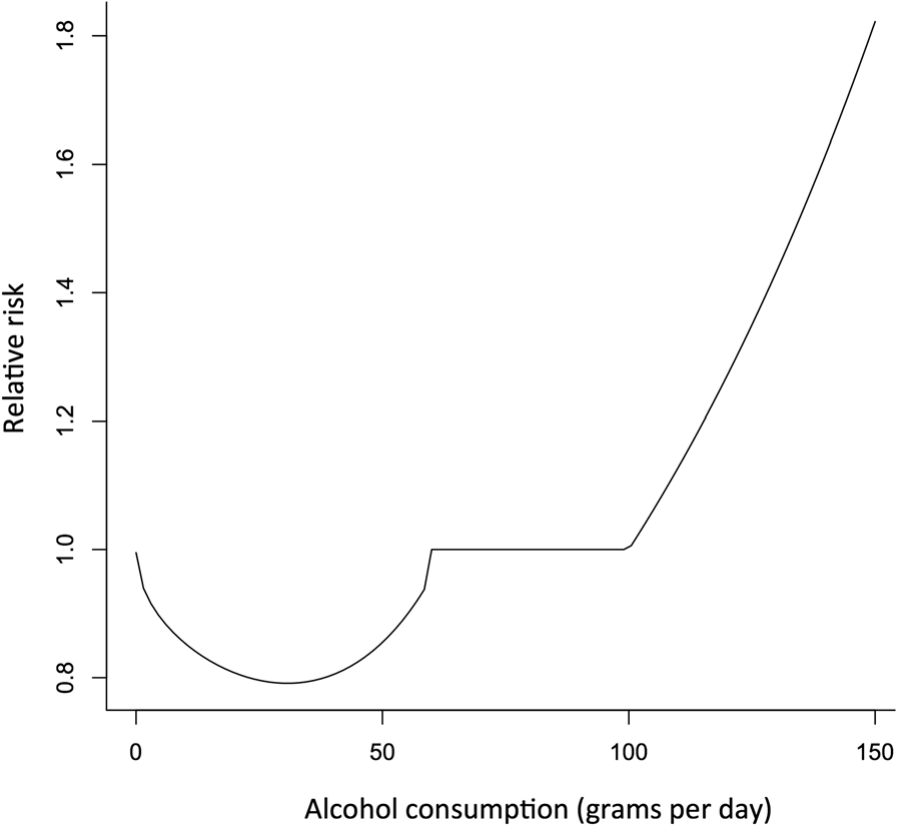
Relative risk function for male drinkers for IHD, all ages combined, using population data from China

**Fig. 2:**
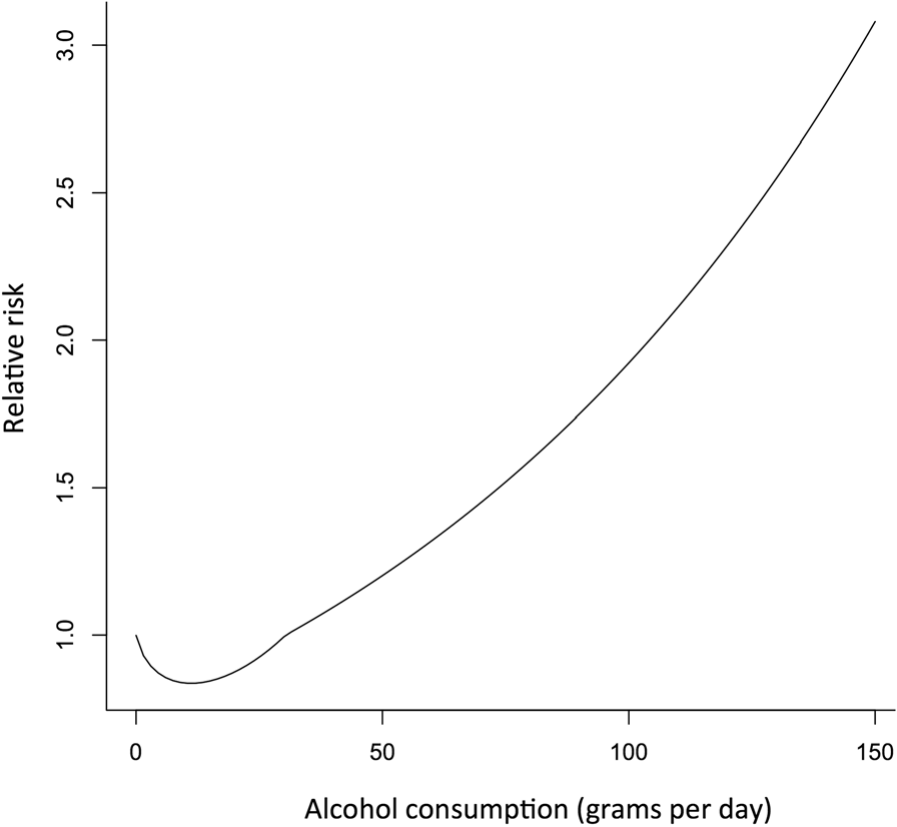
Relative risk function for female drinkers for IHD, all ages combined, using population data from China

Figures 1 and 2 present the new risk relations for IHD for men and women, respectively. These graphs take into account the various RRs by range of underlying exposures found in meta-analyses (see Table 1 for details).

### Resulting alcohol-attributable global mortality burden from ischaemic diseases

Table 2 provides an overview of the recalculated burden for alcohol-attributable ischaemic diseases by region and globally. Russia and surrounding countries are presented as a separate category, as the burdens for these countries were calculated based on country-specific risks (see [35], and above). Overall, the new risk relations resulted in a lower estimated burden, which was driven mainly by a lower estimated burden for women. Proportionally, the effect was substantial, and about the same for IHD and IS; the newly estimated burden for female mortality due to alcohol-attributable ischaemic diseases is approximately one-half of what had been previously estimated (Table 3). The specific contribution of Russia and surrounding countries to the global burden is apparent: for men and the risk of IHD, and for women and the risk of IS, the global alcohol-attributable burden of ischaemic diseases would be negative (i.e., exhibiting a beneficial effect) if results from Russia and surrounding countries were not included.

**Table 2:** Alcohol-attributable ischaemic mortality by sex and WHO region for 2012^a^

Disease and region		Men		Women		Total	
		Deaths	95 % CI	Deaths	95 % CI	Deaths	95 % CI
Ischaemic heart disease							
	Africa	−3,306	(−9,609 to 2,997)	2,302	(−1,800 to 6,405)	−1,004	(−8,525 to 6,517)
	Americas	−2,433	(−27,910 to 23,043)	3,132	(−12,914 to 19,178)	699	(−29,410 to 30,808)
	Eastern-Mediterranean	1,211	(−535 to 2,957)	1,511	(728 to 2,295)	2,722	(808 to 4,636)
	Europe without Russia and surrounding countries^b^	910	(−35,567 to 37,387)	9,290	(−15,998 to 34,578)	10,200	(−34,185 to 54,586)
	Russia and surrounding countries^b^	72,656	(21,148 to 124,164)	99,080	(37,263 to 160,896)	171,736	(91,193 to 252,278)
	South-East Asia	−1,377	(−23,721 to 20,966)	10,710	(4,519 to 16,900)	9,333	(−13,852 to 32,518)
	Western-Pacific	−13,046	(−67,728 to 41,637)	23,919	(−15,270 to 63,107)	10,873	(−56,402 to 78,148)
	Total^c^	54,499	(22,551 to 86,446)	150,121	(122,037 to 178,206)	204,620	(162,064 to 247,176)
Ischaemic stroke							
	Africa	1,037	(−848 to 2,922)	−640	(−3,450 to 2,171)	397	(−2,987 to 3,782)
	Americas	3,375	(−3,755 to 10,506)	−5,626	(−12,292 to 1,040)	−2,251	(−12,012 to 7,511)
	Eastern-Mediterranean	871	(−581 to 2,323)	132	(−769 to 1,034)	1,003	(−706 to 2,712)
	Europe without Russia and surrounding countries^b^	8,691	(−5,981 to 23,363)	−9,399	(−24,203 to 5,405)	−708	(−21,551 to 20,135)
	Russia and surrounding countries^b^	22,374	(11,573 to 33,174)	46,155	(34,239 to 58,072)	68,529	(52,467 to 84,591)
	South-East Asia	7,264	(−1,694 to 5,501)	1,766	(−5,836 to 9,368)	9,030	(−394 to 18,453)
	Western-Pacific	20,239	(−2,534 to 43,011)	−9,284	(−40,890 to 22,322)	10,954	(−28,001 to 49,910)
	Total^c^	64,040	(53,211 to 74,868)	23,042	(9,481 to 36,602)	87,082	(69,730 to 104,443)

^a^For definitions of WHO regions, see Additional file 1: Web Appendix 2

^b^Russia and its surrounding countries (Belarus, Moldova, Russia and Ukraine)

^c^Total is more than the sum of the regions because of non-WHO member states

**Table 3:** Global alcohol-attributable deaths from cardiovascular diseases in 2012 from the 2014 GSRAH [19] and based on the latest meta-analyses

Disease		Men	Women	Total
	Conduction disorder and other dysrhythmias	7,373	7,835	15,208
	Hypertension	70,051	24,664	94,714
	Haemorrhagic stroke	245,930	132,828	378,757
	Ischaemic heart disease	54,499	150,121	204,620
	Ischaemic stroke	64,040	23,042	87,082
	Total CVD current analysis	441,893	338,490	780,381
*From* [19]				
	*Ischaemic heart disease*	*111,755*	*417,469*	*529,225*
	*Ischaemic stroke*	*64,390*	*45,979*	*110,369*
	*Total CVD*	*499,499*	*628,775*	*1,128,273*

### Impact on the global alcohol-attributable mortality burden from CVDs

Table 3 outlines the estimated burden of CVDs attributable to alcohol using the old and new methodologies. Using the new methodology, alcohol-attributable CVDs accounted for 1.4 % of all deaths (1.5 % for men and 1.3 % for women) and for 26.6 % of all alcohol-attributable deaths (20.1 % for men and 45.7 % for women). The percentages obtained using the new methodology differ from the 2014 GSRAH estimates [19] by−0.6 % with respect to all deaths (−0.2 % for men and−1.1 % for women) and by−10.6 % with respect to all alcohol-attributable deaths (−2.6 % for men and−28.2 % for women).

## Discussion

### Statement of principal findings

We used the most recent meta-analyses to estimate the global mortality burden for 2012, and found marked differences in the burden for both IHD and IS, with the new results being 348,000 deaths lower globally (primarily in women) compared to current WHO GSRAH estimates. However, the overall impact of alcohol consumption on CVD is still detrimental, with approximately 780,000 deaths due to CVD attributable to alcohol consumption globally. While this article is restricted to CVD outcomes, it should be stated, however, that the overall detrimental effect of alcohol is mainly via cancer, alcohol use disorders, liver cirrhosis, and unintentional and intentional injury [19].

### Strengths and weaknesses of the study

In our revision, we used the most recent meta-analyses [21–23] and restricted ourselves to the data range of these analyses to avoid problems based on just extrapolating trends which is especially problematic for exponential trends. A further strength of this strategy is that all of the studies to date were included in a systematic fashion. However, the results of all systematic reviews and meta-analyses can only be as good as the underlying data, and in the field of alcohol epidemiology, there is still a scarcity of well conducted studies which separate the effects of different patterns of drinking (for listing of relevant studies see [22, 46]; for the general question see [49]). Thus, while the proposed methodology is based on the best available evidence systematically collected, there are still many holes in the literature on the impact of different drinking pattern on CVD outcomes.

Second, we set the RR (compared to lifetime abstainers) to at least 1 if people had irregular or regular heavy drinking occasions, which is justified by the underlying recent meta-analyses. Finally, we used disease-specific RRs for former drinkers (i.e., sex-specific RRs for IHD and IS from [36, 37]), and capped the prevalence of former drinkers in regions where sick quitters are less likely (e.g., in regions where lifetime abstention is the norm and where many former drinkers likely drank only for a short period of time). The capping of the prevalence of former drinkers was determined to be the most important factor contributing to the differences between the 2014 GSRAH estimates and the estimates provided in this paper. Clearly, more research is necessary on former drinkers in low- and middle income countries, which will hopefully inform further iterations of the As a result, it is likely that the estimates presented in this paper are a more accurate representation of the burden of alcohol consumption than are the 2014 GSRAH estimates.

The exposure data from the 2014 GSRAH have been validated by experts in the respective countries (for detailed description of the procedures used: [27]; see also [50]), and are based on a triangulation of *per capita* consumption with survey data [51, 52]. While this procedure cannot eliminate all biases, especially in countries with high unrecorded consumption [53, 54], it does correct for errors and biases stemming from survey-based data (for reasoning, see [51, 52]; for estimating uncertainty, see [55]). In other words, the underestimation of consumption via surveys, where in some countries like Canada the survey responses covered less than 35 % of the *per capita* consumption which is mostly based on taxation ([56]; general discussion in [51, 52]), could be avoided. However, as indicated above, unrecorded consumption may contain considerable measurement bias [53, 54].

Mortality data are mainly based on verbal autopsies, especially in low-income countries, and while they certainly contain errors, they are the best available current data [57]. Lastly, the new RR estimates used in this study were based on a larger pool of studies, and thus constitute an improvement. In addition, we were able to base alcohol burden estimates on disease- and sex-specific meta-analyses, and to use age-specific relative risk functions.

### Strengths and weaknesses in relation to other studies, discussing important differences in results

As indicated above, this study is the only one to date which incorporated the latest evidence from systematic reviews and meta-analyses. Thus, it constitutes the best effort to date to model the impact of alcohol on CVDs. All prior efforts [11, 18, 58] used the same principal methodology, but employed less comprehensive meta-analyses on risk relations, and fewer systematic searches for exposure data.

### Meaning of the study

The best possible estimate of the impact of alcohol on CVD mortality revealed an important global detrimental effect. A relatively large proportion of these deaths stem from Russia and surrounding countries, with their specific combination of overall high volume of alcohol consumption coupled with prolonged binges [34, 35, 59–61]. Accordingly, policy makers should act to reduce overall alcohol consumption as well as heavy drinking occasions in order to reduce the alcohol-attributable burden of disease in general and the alcohol-attributable non-communicable disease burden in particular [62].

### Further unanswered questions and future research

There is an ongoing debate as to whether the beneficial effects of alcohol consumption in terms of a protective effect on ischaemic diseases are real. The newest contribution to this discussion comes from Mendelian randomization studies [63, 64], where Holmes and colleagues [63] recently investigated the role of alleles of the ADH1B gene on IHD risk. The rs1229984 A-allele has been found to lead to an increased metabolization of alcohol into acetaldehyde [65] (acetaldehyde is associated with negative effects, including a flushing response). Holmes and colleagues showed that carrying the rs1229984 A-allele was associated with less consumption in terms of both average alcohol consumption and binge drinking, and with a lower IHD risk. While Holmes and colleagues concluded that there is no beneficial effect of alcohol consumption on IHD risk, their findings do not rule out a beneficial effect of low average alcohol consumption without binge drinking. However, the findings highlight the need for careful interpretation of the evidence. Meta-analyses of high quality observational studies, supported by short-term experimental studies on biomarkers for IHD risk, identified a curvilinear relationship between average alcohol consumption and IHD risk, and a modifying effect from episodic heavy drinking (see also [66]). We would expect on average low or moderate alcohol consumption coupled with no binge drinking to show less IHD risk compared to other drinkers. If this rather complex relationship is not reflected in the analysis, interpretation becomes increasingly difficult. Moreover, certain assumptions must be met in order to assess the validity of the findings from a Mendelian randomization analysis, and the conclusions of such an analysis depend on these assumptions. First, it was assumed that all of the effect of the allele was mediated through alcohol consumption. However, only a small percentage of the variation of alcohol consumption is explained by ADH1B genotypes, or by other genes related to alcohol consumption, such as ALDH2. Furthermore, it is assumed that the risk for IHD is identical for carriers and non-carriers, except for the effect of the allele. Additionally, the effect of the allele, other than a reduction in alcohol exposure, must be independent from IHD risk. However, Holmes and colleagues demonstrated that the rs1229984 A-allele was associated with several IHD risk factors. Another problem lies in the statistical power required to examine such a complex association. Only a small percentage of the population studied by Holmes and colleagues carried the rs1229984 A-allele and, thus, investigations in populations where carriers are more common might remedy this problem. While Mendelian randomization has theoretical advantages over observational studies, it is unknown whether such an investigation of the ADH1B gene meets the assumptions necessary to yield unbiased results, particularly in more complex analyses. More research on the influence of several different genes on alcohol consumption and their influence on IHD risk is necessary to validate and correctly interpret the findings from this and other Mendelian randomization studies.

The above discussion illustrates that the relationship between alcohol and CVDs is still very much under examination. However, the new data presented in this article reflect the current state of knowledge and are based on a more robust evidence base than any prior estimates. Thus, unless the knowledge base changes substantially, these new estimates can be considered the best estimates for modelling the impact of alcohol consumption on CVDs.

## Conclusions

When the most comprehensive and recent systematic reviews and meta-analyses are taken as bases and when two dimensions of alcohol consumption are included, the net impact of alcohol consumption on CVD is lower than previously estimated.

### Ethics approval and consent to participate

Secondary analyses of published data. The study was approved by the Research Ethics Board of the Centre for Addiction and Mental Health, Toronto, Canada, as part of the Comparative Risk Analyses for alcohol.

### Consent for publication

Not applicable.

### Availability of data and materials

We used only publicly available data as specified in the manuscript. The R programs to program the AAFs used can be obtained from the first author (jtrehm@gmail.com).

## Additional file

Additional file 1: Web appendix 1. Uncertainty. Web appendix 2. Algorithms for the prevalence of former drinkers. Web appendix 3. Countries in each World Health Organization region. Web appendix 4. Estimating age-specific risk relations for Russia and surrounding countries. (DOCX 22 kb)

## Abbreviations

AAF: alcohol-attributable fractions
CVD: cardiovascular disease
GISAH: Global Information System on Alcohol and Health
GSRAH: Global Status Reports on Alcohol and Health (from WHO)
IHD: ischaemic heart disease
IS: ischaemic stroke
RR: relative risk
WHO: World Health Organization
