# Supplementary material for: Modelling the impact of alcohol consumption on cardiovascular disease mortality for comparative risk assessments: an overview
Source: BMC Public Health. 2016 Apr 28;16:363. doi: 10.1186/s12889-016-3026-9 (PMC4848866; doi:10.1186/s12889-016-3026-9)
Supplement: Additional file 1: — Web appendix 1. Uncertainty. Web appendix 2. Algorithms for the prevalence of former drinkers. Web appendix 3. Countries in each World Health Organization region. Web appendix 4. Estimating age-specific risk relations for Russia and surrounding countries. (DOCX 22 kb) [file 12889_2016_3026_MOESM1_ESM.docx]

## Web appendix 1. Uncertainty

To compute uncertainty for the alcohol-attributable fractions (AAFs), we did following steps.

After calculating the sex-, age- and cause of death-specific AAFs, we estimated their variances. The AAFs as described in the main article depend on the relative risk function, the prevalence of former drinkers and abstainers, and the distribution of consumption among drinkers. Since measurement errors in these values and functions are non-trivial, it is virtually impossible to compute the variance of AAFs algebraically. Thus we used Monte Carlo methodology by generating random sets of all the parameters. In a methodological study, it was determined that 150,000 samples yielded sufficiently accurate confidence intervals. More details can be found in [1].

### Reference List

1. Gmel GJ, Shield KD, Frick H, Kehoe T, Gmel G, Rehm J: **Estimating uncertainty of alcohol-attributable fractions for infectious and chronic diseases**. *BMC Med Res Methodol* 2011, **11**:48.

## Web appendix 2. Algorithms for the prevalence of former drinkers

The following table lists the rules applied to the percentage of former drinkers provided.

| Region* | Men | Women |
| --- | --- | --- |
| AFR | Former drinkers are capped at 5% (the remaining become lifetime abstainers; LTA) | Former drinkers are capped at 5% (the remaining become abstainers) |
| AMR | - USA & Canada: Former drinkers not capped.  - all other countries capped at 5% (the remaining become LTA) | - USA & Canada: Former drinkers capped at 10%  - all other countries capped at 5% (the remaining become LTA) |
| EMR | Former drinkers are capped at 5% (the remaining become LTA) | Former drinkers are capped at 5% (the remaining become LTA) |
| EUR (except Russia and surrounding countries)** | No change applied | Former drinkers capped at 10% (the remaining become LTA) |
| SEAR | Former drinkers are capped at 5% (the remaining become LTA) | Former drinkers are capped at 5% (the remaining become LTA) |
| WPR | - China, Japan, Australia, New Zealand, South Korea, Singapore, Brunei: Former drinkers capped at 10%  - all other countries are capped at 5% (the remaining become lifetime LTA) | - China, Japan, Australia, New Zealand, South Korea, Singapore, Brunei: Former drinkers capped at 10%  - all other countries are capped at 5% (the remaining become LTA) |
| NON-MS | Former drinkers capped at 10% (the remaining become LTA) | Former drinkers capped at 10% (the remaining become LTA) |

* African Region (AFR), Region of the Americas (AMR), South-East Asia Region (SEAR), European Region (EUR), European Region (EUR), Western Pacific Region (WPR), Countries that are outside of the WHO regions (NON-MS).

** Russia and surrounding countries includes Belarus, Moldova, Russia and Ukraine.

## Web appendix 3. Countries in each World Health Organization region

**African Region (AFR):** Algeria, Angola, Benin, Botswana, Burkina Faso, Burundi, Cabo Verde, Cameroon, Central African Republic, Chad, Comoros, Congo, Côte d'Ivoire, Democratic Republic of the Congo, Equatorial Guinea, Eritrea, Ethiopia, Gabon, Gambia, Ghana, Guinea, Guinea-Bissau, Kenya, Lesotho, Liberia, Madagascar, Malawi, Mali, Mauritania, Mauritius, Mozambique, Namibia, Niger, Nigeria, Rwanda, Sao Tome and Principe, Senegal, Seychelles, Sierra Leone, South Africa, Swaziland, Togo, Uganda, United Republic of Tanzania, Zambia, and Zimbabwe

**Region of the Americas (AMR):** Antigua and Barbuda, Argentina, Bahamas, Barbados, Belize, Bolivia (Plurinational State of), Brazil, Canada, Chile, Colombia, Costa Rica, Cuba, Dominica, Dominican Republic, Ecuador, El Salvador, Grenada, Guatemala, Guyana, Haiti, Honduras, Jamaica, Mexico, Nicaragua, Panama, Paraguay, Peru, Saint Kitts and Nevis, Saint Lucia, Saint Vincent and the Grenadines, Suriname, Trinidad and Tobago, United States of America, Uruguay, and Venezuela (Bolivarian Republic of)

**South-East Asia Region (SEAR):** Bangladesh, Bhutan, Democratic People's Republic of Korea, India, Indonesia, Maldives, Myanmar, Nepal, Sri Lanka, Thailand, and Timor-Leste

**European Region (EUR):** Albania, Andorra, Armenia, Austria, Azerbaijan, Belarus, Belgium, Bosnia and Herzegovina, Bulgaria, Croatia, Cyprus, Czech Republic, Denmark, Estonia, Finland, France, Georgia, Germany, Greece, Hungary, Iceland, Ireland, Israel, Italy, Kazakhstan, Kyrgyzstan, Latvia, Lithuania, Luxembourg, Malta, Monaco, Montenegro, Netherlands, Norway, Poland, Portugal, Republic of Moldova, Romania, Russian Federation, San Marino, Serbia, Slovakia, Slovenia, Spain, Sweden, Switzerland, Tajikistan, The former Yugoslav Republic of Macedonia, Turkey, Turkmenistan, Ukraine, United Kingdom, and Uzbekistan

**Eastern Mediterranean Region (EMR):** Afghanistan, Bahrain, Djibouti, Egypt, Iran (Islamic Republic of), Iraq, Jordan, Kuwait, Lebanon, Libya, Morocco, Oman, Pakistan, Qatar, Saudi Arabia, Somalia, South Sudan, Sudan, Syrian Arab Republic, Tunisia, United Arab Emirates, and Yemen

**Western Pacific Region (WPR):** Australia, Brunei Darussalam, Cambodia, China, Cook Islands, Fiji, Japan, Kiribati, Lao People's Democratic Republic, Malaysia, Marshall Islands, Micronesia (Federated States of), Mongolia, Nauru, New Zealand, Niue, Palau, Papua New Guinea, Philippines, Republic of Korea, Samoa, Singapore, Solomon Islands, Tonga, Tuvalu, Vanuatu, and Viet Nam

## Web appendix 4. Estimating age-specific risk relations for Russia and sourounding countries

The age adjustment of the alcohol Relative Risk (RR) functions for ischemic heart disease Belarus, Moldova, Russia and the Ukraine was performed using the RR functions from [[1](#_ENREF_1)] and the age RR ratios from [[2](#_ENREF_2)]. The age adjusted RR was calculated using the following formula:

$$RRi(x)AA=\exp\left( AgeAdjust \right)*exp(\ln\left( RRi(x) \right))$$

where ln*RRi(x)* represents the natural logarithm of the RR for disease *i* and alcohol consumption *x* [[1](#_ENREF_1)], *AgeAdjust* is the age adjustment coefficient (different coefficients were used for the ages 15 to 34, 35 to 64, and 65 plus) and RR*i(x)AA* represents the age-adjusted RR for disease *i* and consumption *x*.

AgeAdjust coefficients were estimated based on the ratio of the RR*i(x)* between people aged 35 to 54 years and aged 55 to 74 years. This ratio was transformed using the natural logarithm and divided by the group age difference to obtain the natural logarithmic ratio increase by age. The AgeAdjust coefficients for those people 15 to 34, 35 to 64, and 65 years of age and older were estimated using the logarithmic relationship and the difference between the mean age of the age group of interest and the mean age of death upon which the original RRs were based, using data from [[1](#_ENREF_1)].

The uncertainty for the AgeAdjust coefficients was calculated based on 40,000 simulations. The age-specific RRs were provided for current smokers by the following alcohol consumption categories: <1 bottle of vodka, 1 to <3 bottles of vodka, and 3 or more bottles of vodka, and no specific sample size data were provided for these groups. Therefore, imputation was performed using the sample size for male current smokers and the total male sample population within each alcohol consumption risk group, and the total male sample population in each age group

The age adjustment method presented here differs from the method used for all other countries as a large proportion of the uncertainty distributions for the RRs for ischemic heart disease from [[2](#_ENREF_2)] crossed zero. Since this uncertainty distribution crossed zero, the variance of x/y was not interpretable especially for areas of the probability distribution when y approaches 0 as the ratio will approach positive or negative infinity and is then undefined at 0. Additionally the age adjustment for ischemic stroke using data from [[2](#_ENREF_2)] was found to be not significant and thus no age adjustment was performed for ischemic stroke for Russia and the surrounding countries. Therefore, more research is needed to determine if age adjustment for the alcohol RR for ischemic stroke is needed.

### Reference List

1. Zaridze D, Brennan P, Boreham J, et al. **Alcohol and cause-specific mortality in Russia: a retrospective case-control study of 48,557 adult deaths**. *Lancet* 2009;373:2201-14.

2. Zaridze D, Lewington S, Boroda A, et al. **Alcohol and mortality in Russia: prospective observational study of 151,000 adults**. *Lancet* 2014;pii: S0140-6736:62247-3.
